# Supplementary material for: Gender Differences of the Effect of Vaccination on Perceptions of COVID-19 and Mental Health in Japan
Source: Vaccines (Basel). 2023 Apr 10;11(4):822. doi: 10.3390/vaccines11040822 (PMC10143812; doi:10.3390/vaccines11040822)
Supplement: Supplementary file 1 [file vaccines-11-00822-s001.zip › vaccines-2235647-supplementary.pdf]

vaccines MDPI

Table S1. FE model: Dependent variables are perceptions of COVID-19 and mental health. Male sample. (Alternative specification).

	(1) *PROB_ COVID19*	(2) *SEVER_ COVID19*	(3) *HAPPY*	(4) *FEAR*	(5) *ANXIETY*	(6) *ANGER*
VACCINE FIRST	−1.876 ***	−0.015	0.0003	0.003	0.008	0.010
VACCINE SECOND_1	−4.23 ***	−0.167 ***	−0.005	−0.034	−0.042	0.014
Adj R^2	0.57	0.65	0.77	0.56	0.57	0.57
Obs.	27,316	27,316	27,316	27,316	27,316	27,316

Note: The set of control variables used in Table 4 is included, although the results are not reported. ***$p < 0.01$, **$p < 0.05$, *$p < 0.10$.

Table S2. FE model: Dependent variables are perceptions of COVID-19 and mental health. Female sample. (Alternative specification).

	(1) *PROB_ COVID19*	(2) *SEVER_ COVID19*	(3) *HAPPY*	(4) *FEAR*	(5) *ANXIETY*	(6) *ANGER*
VACCINE FIRST	−0.624	−0.074 **	0.049	−0.084 ***	−0.059 *	−0.029
VACCINE SECOND_1	−5.151 ***	−0.174 ***	0.127 **	−0.134 ***	−0.085 ***	−0.021
Adj R^2	0.56	0.68	0.73	0.54	0.55	0.48
Obs.	26,691	26,691	26,691	26,691	26,691	26,691

***$p < 0.01$, **$p < 0.05$, *$p < 0.10$.

Table S3. FE model: Dependent variables are perceptions of COVID-19 and mental health. Simpler model where VACCINE is replaced by VACCINE FIRST and VACCINE SECOND_1.

	(1) *PROB_ COVID19*	(2) *SEVER_ COVID19*	(3) *HAPPY*	(4) *FEAR*	(5) *ANXIETY*	(6) *ANGER*
Full sample of (Table 4)						
VACCINE	−3.273 ***	−0.118 ***	0.044 **	−0.066 ***	−0.047 **	−0.006
Sub-sample of males (Table 7)						
VACCINE	−3.274 ***	−0.105 ***	−0.003	−0.019	−0.021	−0.012
Sub-sample of females (Table 8)						
VACCINE	−3.290 ***	−0.133***	0.094 ***	−0.114 ***	−0.074 ***	−0.024

***$p < 0.01$, **$p < 0.05$, *$p < 0.10$.
